# *AIF-1* gene does not confer susceptibility to Behçet’s disease: Analysis of extended haplotypes in Sardinian population

**DOI:** 10.1371/journal.pone.0204250

**Published:** 2018-09-25

**Authors:** Maria Maddalena Angioni, Matteo Piga, Fabiana Paladini, Sara Lai, Gian Luca Erre, Alberto Floris, Alberto Cauli, Maria Teresa Fiorillo, Giuseppe Passiu, Carlo Carcassi, Rosa Sorrentino, Alessandro Mathieu

**Affiliations:** 1 Chair of Rheumatology and Rheumatology Unit, University and AOU of Cagliari, Cagliari, Italy; 2 Department of Biology and Biotechnology “C. Darwin”, Sapienza University of Rome, Rome, Italy; 3 Chair of Medical Genetics, University of Cagliari, Cagliari, Italy; 4 Chair of Rheumatology and Rheumatology Unit, University and AOU of Sassari, Sassari, Italy; Illumina Inc, UNITED STATES

## Abstract

**Background:**

Behçet’s disease (BD) is a polygenic immune-mediated disorder characterized by a close association with the HLA-B*51 allele. The HLA region has a strong linkage disequilibrium (LD) and carries several genetic variants (e.g. *MIC-A*, *TNF*-α genes) identified as associated to BD because of their LD with HLA-B*51. In fact, the HLA-B*51 is inherited as part of extended HLA haplotypes which are well preserved in patients with BD. Sardinian population is highly differentiated from other Mediterranean populations because of a distinctive genetic structure with very highly preserved HLA haplotypes.

**Patients and methods:**

In order to identify other genes of susceptibility to BD within the HLA region we investigated the distribution of human Allograft Inflammatory Factor-1 (*AIF-1*) gene variants among BD patients and healthy controls from Sardinia. Six (rs2736182; rs2259571; rs2269475; rs2857597; rs13195276; rs4711274) *AIF-1* single nucleotide polymorphisms (SNPs) and related extended haplotypes have been investigated as well as their LD within the HLA region and with HLA-B*51. Overall, 64 BD patients, 43 HLA-B*51 positive healthy controls (HC) and 70 random HC were enrolled in the study.

**Results:**

HLA-B*51 was the only allele with significantly higher frequency (*p*_*c*_ = 0.0021) in BD patients (40.6%) than in HC (9.8%). The *rs2259571*^T^ *AIF-1* variant had a significantly reduced phenotypic, but not allelic frequency in BD patients (72.1%; p_c_ = 0.014) compared to healthy population (91.3%). That was likely due to the LD between HLA-B*51 and *rs2259571*^G^ (*p*_*c*_ = 9E-5), even though the *rs2259571*^G^ distribution did not significantly differ between BD patients and HC.

**Conclusion:**

No significant difference in distribution of *AIF-1* SNPs haplotypes was observed between BD patients and HC and between HLA-B*51 positive BD patients and HLA-B*51 positive HC. Taken together, these results suggest that *AIF-1* gene is not associated with susceptibility to BD in Sardinia.

## Introduction

Behçet’s disease (BD) is a chronic vasculitis characterized by recurrent oral ulcers, genital ulcers, ocular and skin manifestations with involvement of arteries and veins of all sizes. BD clusters in an area between latitudes 30° N and 45° N spanning from the far Eastern Asia to the Mediterranean basin [1,2]. Such a distinctive clustering seems related to geographical distribution of genetic susceptibility factors among general population [3,4]. As described in detail previously [5,6], several lines of evidence suggest that host genetic factors play a pivotal role in determining susceptibility to BD and its close association with the HLA-B*51 allele represents the clearest evidence of a genetic contribution to the disease. However, HLA-B*51 alone is neither necessary nor sufficient to BD development and other susceptibility genes, whose products are responsible for inflammatory and immune-mediated mechanisms, have been identified both outside and within the HLA region [6–8]. Genome wide association studies (GWAS) identified a strong association between BD and the HLA region comprehensive of HLA-B*51 within an extended haplotype [9]. These findings were suggestive of the presence of additional genes within the HLA region conferring susceptibility to BD. Actually, additional genes within the region such as other HLA class I alleles (e.g. B*15, B*57, A*26), the *TNF*-α and the MHC Class I chain-related gene A (*MIC-A*) have been associated to an increased risk of BD [10–12].

We previously pointed out that [13], in Sardinia, the BD-associated HLA-B*51:01 allele is inherited as part of a haplotype which is different from that characterizing the B*51:01-positive healthy controls. The HLA haplotype distribution in Sardinians, compared to other Mediterranean populations, is characterised by a small number of preserved and highly frequent haplotypes and by a very high number of rare haplotypes [14,15]. Therefore, the peculiar genetic background of Sardinians represents a valuable source for studying HLA-related genetic and epigenetic associations to BD [16].

Human Allograft Inflammatory Factor-1 (*AIF-1*) is a 143 amino acid, 17 kDa, cytoplasmic calcium-binding protein, encoded within the HLA class III region on chromosome 6p21 which is densely clustered with genes involved in the inflammatory responses including *TNF-α*. Because its pro-inflammatory role, *AIF-1* is involved in various inflammatory pathological processes such as allograft rejection, autoimmune diseases, inflammatory central nervous system injury. Several single-nucleotide polymorphisms (SNPs) have been identified in the *AIF-1* gene as associated with autoimmune diseases [17,18]. Considering its position within the HLA region, between the *TNF*-α gene promoter and the HLA-B locus, and its pro-inflammatory activity we deemed interesting to study *AIF-1* as a candidate gene for BD susceptibility.

The objective of this study was to investigate the association of selected *AIF-1* SNPs with susceptibility to BD in Sardinian and their distribution within distinct HLA extended haplotypes harbouring the HLA-B*51 allele.

## Materials and methods

### Patients and controls

Overall, 64 unrelated consecutive Sardinian patients with BD referring to the Rheumatology Unit of Cagliari and classified according to the 1990 International Study Group criteria, were enrolled in this study between January 2014 and December 2016 (Table 1).

**Table 1:** Cumulative features of patients suffering from Behçet’s disease enrolled in this study.

Features	N (%)
**Gender**	F/M = 2/1
**Age at diagnosis** (mean ± SD)	31.0 ± 9.7
**Oral ulcers**	64 (100)
**Genital ulcers**	41 (64)
**Cutaneous involvement**	36 (56.2)
**Ocular involvement**	33 (51.4)
**Neurological involvement**	6 (9.4)
**Vascular involvement**	14 (21.8)
**Musculoskeletal involvement**	21 (32.8)
**Pathergy test positive**	7 (10.9)

Unless otherwise specified, numbers are absolute values, number in brackets are percentage.

Overall, 43 consecutive HLA-B*51 positive and 70 unselected healthy bone marrow donors served as controls. All patients and controls, matched for gender, came from various areas of Sardinia and were representative of the islander population distribution. Both patients and controls gave their written informed consent to the study which protocol was specifically approved by the local ethics committee “Comitato Etico Indipendente AOU Cagliari” (n. 224/CE).

### Genotyping

Peripheral blood from BD patients and HC was drawn in EDTA-containing vials and genomic DNA was extracted using the Nucleic Acid Extraction and Cell Separation Instrument (Manufacturer: DiaSorin Inc.). The amount of DNA was determined using the Qubit fluorometric quantitation that comprises the Qubit 3.0 Fluorometer and sensitive, specific Qubit quantitation DNA assay (Thermo Fisher Scientific). All patients and controls were genotyped for 6 different SNPs of the *AIF-1* gene (Table 2) by the reverse sequence-specific oligonucleotide polymerase chain reaction (PCR) technique using TaqMan SNP Genotyping Assays from Life Technologies according to the manufacturer’s protocol.

**Table 2:** Features of the six SNPs of the *AIF-1* gene typed.

SNP ID	Location	Polymorphism	Molecular Consequences
***rs2736182***	Chr.6: 31615535	A/G, Transition Substitution	2KB upstream variant, missense variant, 5’ UTR variant
***rs2259571***	Chr.6: 31616050	G/T;Transversion substitution	Intron variant 5’ UTR variant
***rs2269475***	Chr.6: 31616154	C/T, Transition Substitution	Intron variant, missense variant
***rs2857597***	Chr.6: 31617223	A/T, Transversion Substitution	500B downstream variant
***rs13195276***	Chr.6: 31616317	C/T, Transition Substitution	intron variant, missense variant
***rs4711274***	Chr.6: 31615389	A/G, Transition Substitution	2KB upstream variant, intron variant

Source: Database of Short Genetic Variation (dbSNP)–NCBI- NIH.

Patients and controls were also typed for HLA-A, B, C, DRB1, DQA1 and DQB1 using commercial kits (HLA SSP kits; Biotest, Dreieich, Germany) in order to identify a different distribution of the *AIF-1* SNPs in the extended HLA haplotypes.

It is well known that choosing preliminary candidate SNPs is critical for candidate gene association studies. The chosen SNPs were based on previously described associations in various immune-mediated diseases [17,18], as well as according to functional features deemed interesting by the authors.

### Statistical analysis

Hardy–Weinberg equilibrium (HWE) was tested using the Chi-square test. To assess differences in the proportions of *AIF-1* polymorphic alleles and disease associations in healthy controls (HC) versus BD patients, chi-square test or two-tailed Fisher’s exact test, for low frequency, were performed using MedCalc software (version 16.8.4, Mariakerke, Belgium). The strength of association was estimated by calculating the odds ratio (OR) with 95% Confidence Interval (95% CI). Under the assumption of independence, a value of p<0.05 was considered statistically significant where Bonferroni correction was applied for multiple comparisons to all novel associations, with a correction factor derived from the number of alleles examined; *p*_*c*_ indicates where the Bonferroni correction was applied. The LD among the 6 SNPs of the *AIF-1* gene and between single SNPs and HLA-B*51 was calculated using the HaploView 4.2 software.

## Results

HLA-B*51 phenotype frequency was significantly higher (*p*_*c*_ = 0.0021; OR = 6.2; 95%CI 2.5 to 15.8) in BD patients (40.6%) than in HC (9.8%). No other HLA class I and II alleles were independently associated with BD.

Six SNPs in *AIF-1* were determined in 64 BD patients, 43 HLA-B*51 positive HC and 70 HC (Table 3).

**Table 3:** Phenotypic frequencies of the 6 *AIF-1* SNPs determined in BD patients and healthy controls according to their HLA-B*51 status.

SNP ID	Genotype/allele	BDn (%)	BD B*51+n (%)	HCn (%)	HC B*51+n (%)	BD vs. HCP_c_
***rs2736182***	G	64 (100)	26 (100)	70 (100)	43 (100)	Ns
	A	5 (7.8)	4 (15.4)	5 (7.1)	2 (4.6)	Ns
	G/G	59 (92.2)	22 (84.6)	65 (92.8)	41(95.3)	Ns
	A/A	0	0	0	0	Ns
	A/G	5 (7.8)	4 (15.4)	5 (7.1)	2 (4.6)	Ns
***rs2259571***	T	44 (72.1)	13 (56.5)	63(91.3)	27 (62.8)	0.014
	G	43 (70.5)	18 (78.3)	43(62.3)	38 (88.4)	Ns
	T/T	18 (29.5)	5 (21.7)	26 (37.7)	5 (11.6)	Ns
	G/G	17 (27.9)	10 (43.5)	6 (8.7)	16 (37.2)	Ns
	T/G	26 (42.6)	8 (34.8)	37 (53.6)	22 (51.2)	Ns
***rs2269475***	C	62 (100)	25 (100)	69 (100)	43 (100)	Ns
	T	5 (8.1)	3 (12.0)	3 (4.3)	4 (9.3)	Ns
	C/C	57 (91.9)	22 (88.0)	66 (95.6)	39 (90.7)	Ns
	T/T	0	0	0	0	Ns
	C/T	5 (8.1)	3 (12.0)	3 (4.3)	4 (9.3)	Ns
***rs2857597***	T	5 (7.8)	3 (11.5)	13 (18.6)	4 (9.3)	Ns
	A	64 (100)	26 (100)	69 (98.6)	43 (100)	Ns
	T/T	0	0	1 (1.4)	0	Ns
	A/A	59 (92.2)	23 (88.5)	57 (81.4)	39 (90.7)	Ns
	A/T	5 (7.8)	3 (11.5)	12 (17.1)	4 (9.3)	Ns
***rs13195276***	C	63 (100)	25 (100)	69 (100)	43 (100)	Ns
	T	63(100)	25 (100)	69 (100)	43 (100)	Ns
	C/C	0	0	0	0	Ns
	T/T	0	0	0	0	Ns
	C/T	63 (100)	25 (100)	69 (100)	43 (100)	Ns
***rs4711274***	G	64 (100)	26 (100)	70 (100)	39 (90.7)	Ns
	A	5 (7.8)	3 (11.5)	3 (4.3)	0	Ns
	G/G	59 (92.2)	23 (88.5)	67 (95.7)	39 (90.7)	Ns
	A/A	0	0	0	0	Ns
	A/G	5 (7.8)	3 (11.5)	3 (4.3)	0	Ns

Numbers are absolute values, number in brackets are percentages. ns: not significant

Five out of 6 *AIF-1* SNPs (rs2736182; rs2269475; rs2857597; rs13195276; rs4711274) did not show different allelic and phenotypic distribution between patients and HC. Only the rs2259571 SNP had a significantly decreased phenotypic, but not allelic, frequency of the *rs2259571*^T^ variant in BD patients (72.1%, Chi squared 9.31, *p*_*c*_ = 0.014) compared to healthy population (91.3%) without a significantly different phenotypic distribution of the *rs2259571*^G^ variant between BD patients and HC despite its LD with the HLA-B*51 (*p*_*c*_ = 9E-5). Noteworthy, the *rs2259571*^T^ phenotypic frequency distribution did not significantly differ between HLA-B*51 positive BD patients (56.5%) and HLA-B*51 positive HC (62.8%).

**Fig 1:**
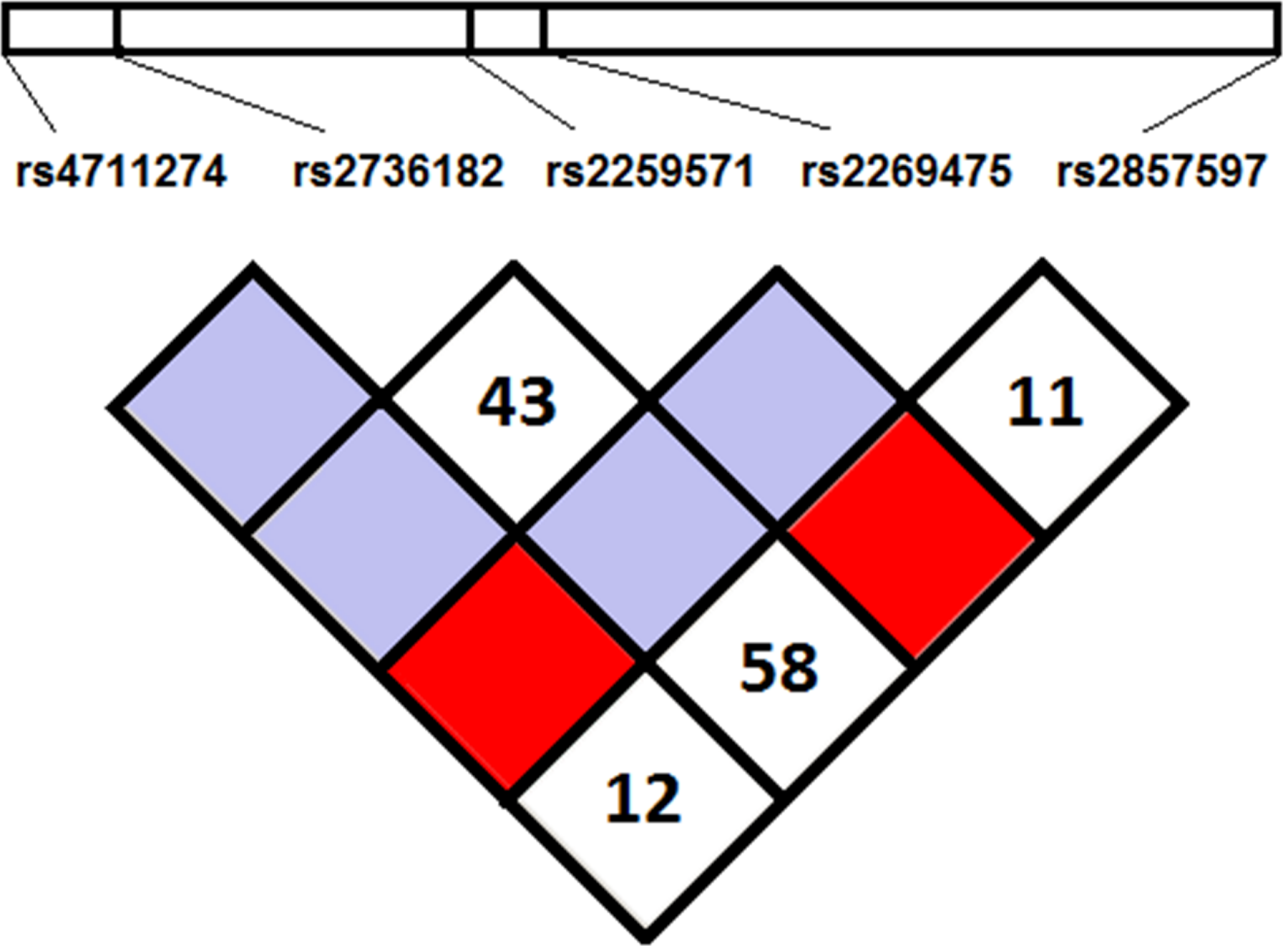
Linkage disequilibrium plot of *AIF-1* SNPs in HLA-B*51 positive and HLA-B*51 negative subjects irrespective of the disease status. The Haploview software automatically omitted rs13195276 SNP from the model because equally expressed in both populations. Values of the pair-wise D’ (multiply by 100) are shown in each white square. 1: rs4711274; 2: rs2736182; 3: rs2259571; 4: rs2269475; 6: rs2857597. Bright red blocks, D′ (normalized linkage disequilibrium measure or D) = 1.0, with logarithm of odds (LOD) score ≥ 2.0; white blocks, D′ < 1.0 with LOD < 2.0; blue blocks, D′ = 1.0 with LOD < 2.0. Numbers in blocks denote D′ values. The genomic organization is described above the LD plot. LOD was defined as log10(L1/L0), where L1 = likelihood of the data under linkage disequilibrium, and L0 = likelihood of the data under linkage equilibrium. D′ was calculated as follows: D′ = (D) divided by the theoretical maximum for the observed allele frequencies.

Analysing the distribution of *AIF-1* SNPs haplotypes, no significantly different haplotype distribution between BD patients and HC was detected (Fig 1). The GGTCA (haplotype frequency 42.0% in BD and 48.0% in HC) and GGGCA (haplotype frequency 47.6% in BD and 35.5% in HC) were the most frequently detected *AIF-1* SNP haplotypes. As effect of the LD between *rs2259571*^*G*^ and HLA-B*51 the GGGCA was found at a higher frequency in HLA-B*51 positive subjects (56.3%) and the GGTCA was most frequently carried by HLA-B*51 negative subjects (52.9%) irrespective of the disease status.

**Fig 2:**
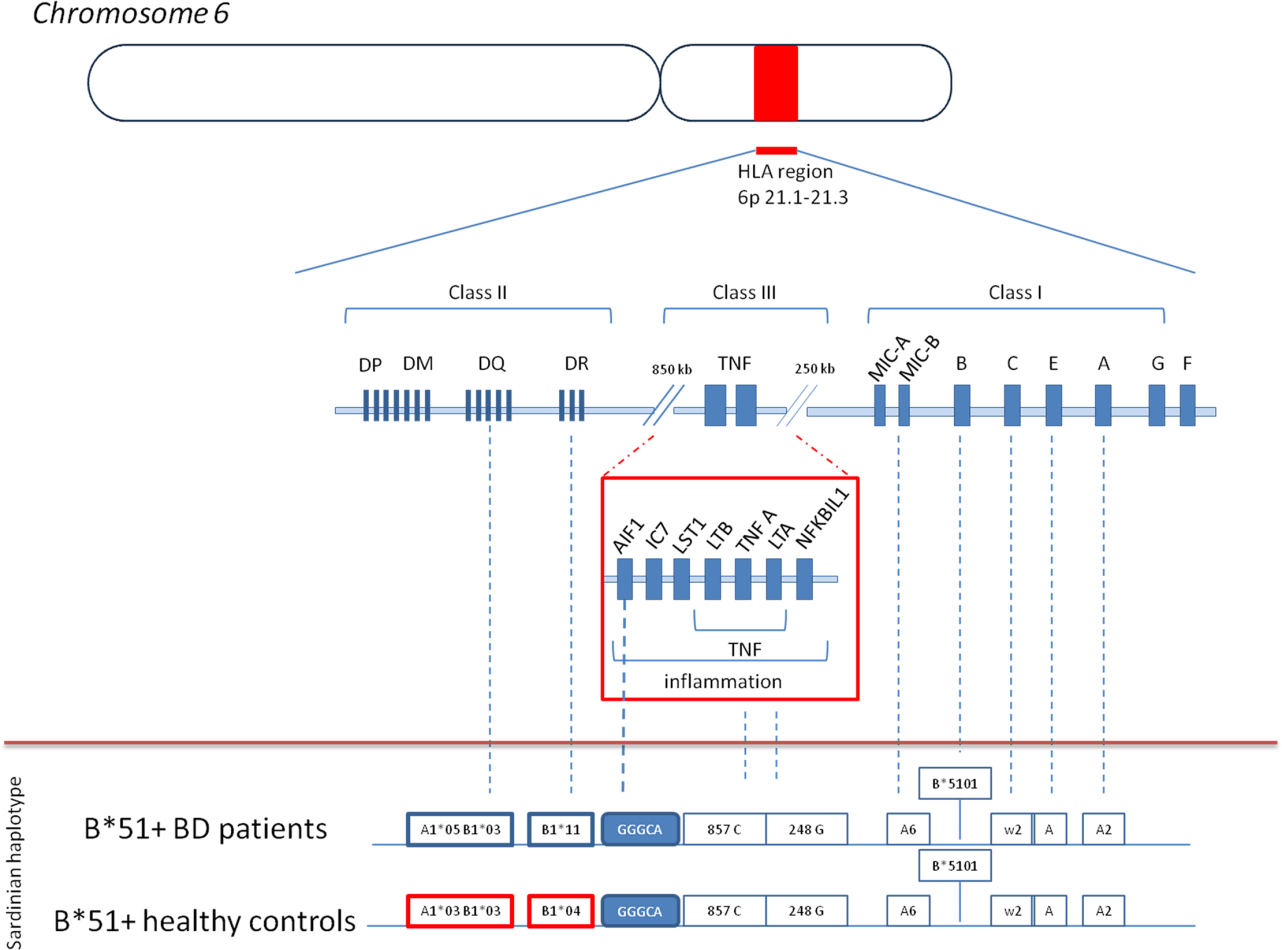
Schematic representation of the HLA region and extended HLA haplotypes harbouring the HLA-B*51 and *AIF-1* alleles in BD patients and healthy controls from Sardinia.

Finally, HLA-B*51 was harboured in two HLA-B-HLA-DR distinct haplotypes: B*51-DR*11 in 14/26 (53.8%) of HLA-B*51 positive BD patients and in 14/37 (37.8%) of HLA-B*51 positive HC (p = 0.231); and B*51-DR*4 in 1/26 (3.8%) of HLA-B*51 positive BD patients and in 14/37 (37.8%) of HLA-B*51 positive HC (p = 0.013; *p*_*c*_ = 0.065 OR 0.06 95%CI 0.01–0.54) according to, but not fully confirming, the previous observation of a lack of association between B*51-DR*4 and BD susceptibility in Sardinians (13). No significant difference in the distribution of *AIF-1* single or combined SNPs was observed between the B*51-DR*11 and B*51-DR*4 extended haplotypes (Fig 2).

## Discussion

The present study firstly reports on the association of six *AIF-1* SNPs with BD susceptibility. The results showed no significant association between each investigated SNP or SNP haplotypes and BD in Sardinia. Despite of the LD between *rs2259571*^G^ and HLA-B*51, the frequency of each SNP and the related GGGCA haplotype was not significantly increased in BD patients. As an effect of the LD with HLA-B*51, the *rs2259571*^G^ was harboured in both B*51-DR*11 and B*51-DR*4 haplotypes, the latter having different distribution in BD patients and HC from Sardinia. These results suggest that polymorphisms of *AIF-1* are not associated with the susceptibility to BD in the Sardinian population.

BD is considered as a complex polygenic disorder with a mixed autoinflammatory and autoimmune pathogenesis [19]. GWAS identified several susceptibility loci associated with BD susceptibility [9,20–23], but always confirmed the major role of the HLA region and especially of the HLA-B*51 allele in BD susceptibility [9,20–23]. Nevertheless, the highest contribution of HLA-B*51 to the overall BD genetic susceptibility was estimated to be only 19% [24]. GWAS also confirmed a strong LD in the HLA region of BD patients, mainly due to the fact that HLA-B*51 was found almost exclusively on a single extended haplotype [9]. In Sardinia, two distinct extended haplotypes harbouring HLA-B*51:01 were identified: A2-Cw2-B*5101-DRB1*11-DQA1*05-DQB1*03, which marks the B*51 positive patients with BD in Sardinia, and A2-Cw2-B*5101-DRB1*04-DQA1*03-DQB1*03, which is significantly more frequent in Sardinian HC than in BD [13]. Considering the high LD in the HLA region, it is conceivable that genes besides HLA-B*51, somewhat involved in the innate and adaptive immune responses and inherited as part of distinct HLA-B*51:01 haplotypes, may play a role in BD susceptibility.

Because of its position within the HLA class III region, between the HLA-B and HLA-DR loci, and its pro-inflammatory effect, the *AIF-1* gene was deemed as a possible determinant of genetic susceptibility to BD. In humans, in fact, AIF-1 influences the immune system at several key points and boosts the expression of inflammatory mediators such as cytokines (IL6, TNF-α), chemokines, inducible nitric oxide synthase and promotes inflammatory cell proliferation and migration [25]. Moreover, AIF-1 is involved in some model of autoimmune diseases such as experimental autoimmune uveitis, encephalomyelitis and neuritis [26,27]. The role of *AIF-1* in rheumatoid arthritis and systemic sclerosis has been investigated and the rs2269475 SNP was found associated with an increased risk of developing both diseases [28,29]. Although preliminary data pointed to a possible role of *AIF-1* in BD susceptibility, we did not find any suggestion for this in our study population.

To the best of our knowledge, this was the first study investigating the role of *AIF-1* in BD. With the aim to elucidate the genetic basis of BD we set a candidate gene case-control association study and we tested six different *AIF-1* SNPs. Major strengths are represented by the peculiar genetic background of Sardinians and by the enrolment of two different control populations allowing to identify a different distribution of *AIF-1* in patients and in controls but also in HLA-B*51 carriers versus other subjects. A major limitation is related to sample size, therefore caution is advised when interpreting results as they may be related to the small size of the population under study.

In conclusion, the present study does not support the hypothesis that a genetically determined regulation of *AIF-1* expression or change in protein structure may predispose to the development of BD in Sardinian patients. Further, larger studies are required to confirm our findings in other populations.

## Supporting information

S1 Table Minimal anonymized dataset. Genotyping results for AIF1 are reported here.
(XLSX)
